# Editorial: Innovations in older adult care and health service management: a focus on China

**DOI:** 10.3389/fpubh.2024.1404227

**Published:** 2024-05-02

**Authors:** Madhan Balasubramanian, Angie A. Shafei, Zhanming Liang

**Affiliations:** ^1^Flinders University, College of Business Government and Law, Healthcare Management and Centre for Social Impact, Adelaide, SA, Australia; ^2^The University of Sydney, Faculty of Medicine and Health, School of Public Health, Menzies Centre for Health Policy and Economics, Sydney, NSW, Australia; ^3^The University of Adelaide, Faculty of Health Sciences, Australian Research Centre for Population Oral Health, Adelaide, SA, Australia; ^4^Flinders University, College of Business Government and Law, Healthcare Management, Adelaide, SA, Australia; ^5^James Cook University, College of Public Health, Medical and Vet Sciences, Australian Institute of Tropical Health and Medicine, Townsville, QLD, Australia

**Keywords:** older adults, aged care, China, health workforce, models of care, health systems

## Introduction

China stands at the forefront of a global demographic shift as home to over 260 million people aged 60 and above – this accounts for one-fifth of the world's total older adult population and standing as the country with the largest number of older adults in the world (1). A recent Lancet editorial on population aging in China argued that the traditional model of care focussed on filial piety is unsustainable for China's rapidly aging population (2). Newer models of care and investments are vital to challenge the emerging crisis of aged care in China. The Lancet Commission report from Peking University identified some of the significant investments made by the Chinese government to support older adults and improve aged care services (3). China is taking massive steps to strengthen institutional and community care infrastructure both as a substitute to and to complement the role of informal carers. Long-term care insurance (LTCI) has been piloted in many cities in China to support older adults, with the government focussing on integration of long-term care with health/hospital care. Given China's unique regional segmentation of health insurance systems and regulatory environment for care provision, the Lancet Commission argued the necessity for better integration of services across regions and occupations, capping LTCI initiatives, expanding services for older adults and striving for a safer regulatory environment.

**Figure 1:**
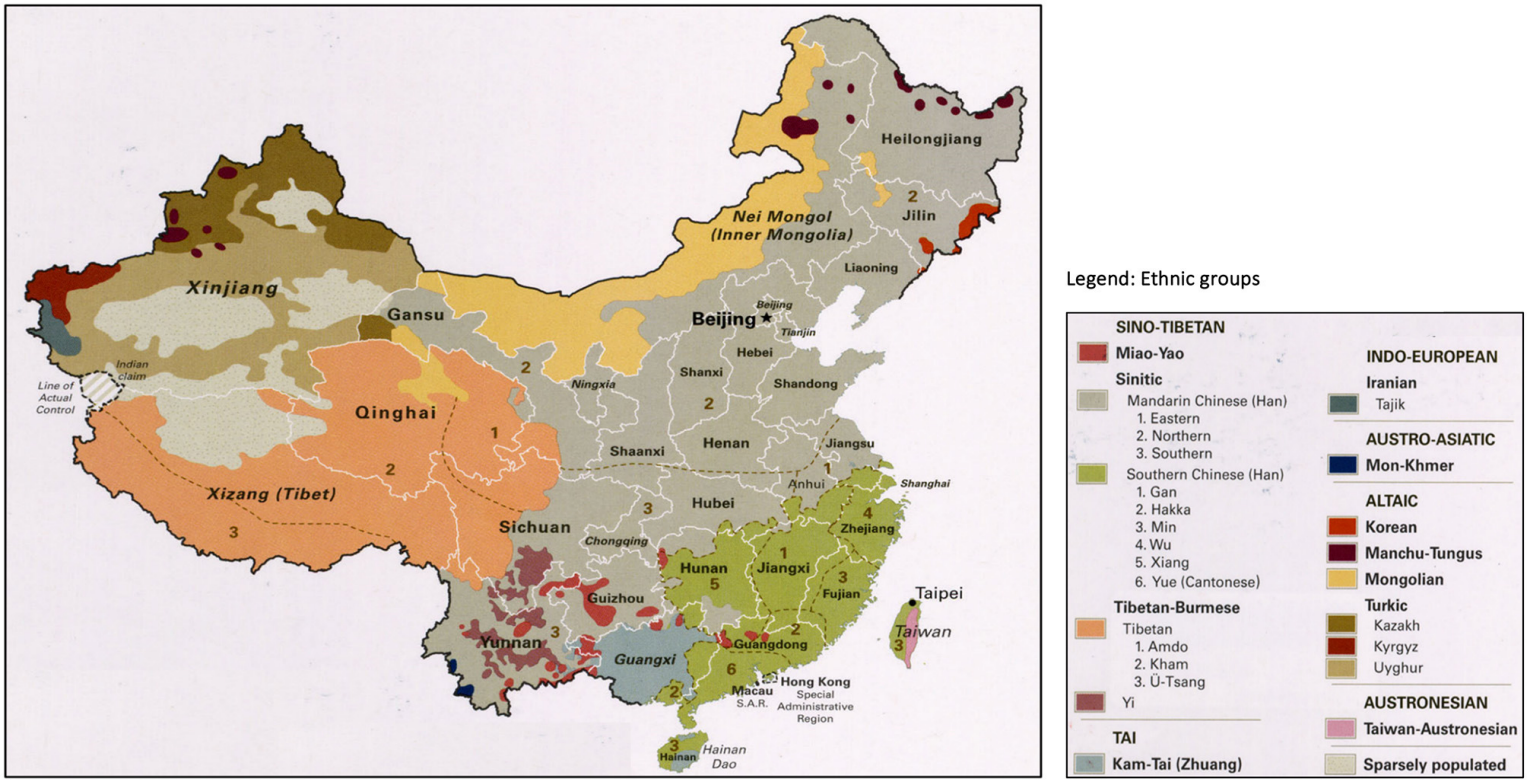
Ethnic and cultural diversity map of China. Source: Adapted from Perry-Castaneda Library Map Collection, Universty of Texas at Austin; Thematic Collection Series. China has 34 administrative dicisions, including 23 provinces, five autonomous regions, four muncipalities and two special administrative regions; China considers Taiwan as its 23rd province.

China's rich cultural heritage and ethnic diversity are influential forces in developing culturally acceptable health care services, particularly for older adults. The Chinese government officially recognizes 56 distinct ethnic groups. The Han Chinese form the majority, accounting for nearly 92% of the population. A large majority of these minority groups are distributed regionally or in rural areas of Xinjiang, Tibet, Inner Mongolia, Guangxi, and Yunnan (see Figure 1). It's vital to note that each ethnic group bring their own beliefs and traditions and diversity introduces unique challenges and opportunities for designing culturally sensitive and accessible models of care. Health workforce issues also contributes to major challenges in providing quality and accessible care (4, 5).

The need for this unique Research Topic stems from the growing number of contributions on aged care innovation and development from China to Frontiers in Public Health. We are grateful for the overwhelming response from our colleagues from China, who shared their excellent research. A total of 127 researchers contributed to this Research Topic. Overall, the issue is already reaching the masses with over 30,000 views, and 6,765 article downloads at the time of writing this editorial. These articles will not only add to the body of knowledge on aged care in China, but also provoke a re-examination of the existing paradigms in aged care research and priorities within China.

## Synopsis of articles in the Research Topic

In this issue we offer a rich and diverse collection of articles coming from the breadth of China. A total of 10 articles included research or evaluation arising from one administrative division, while other articles included research coming from more than one administrative division (see Table 1). A total of 127 researchers from some of the leading academic institutions and health care organizations from China have contributed to the Research Topic.

**Table 1:** Studies in the Research Topic and setting (administrative division).

**Studies with focus on one administrative division**		**Studies with focus on multiple administrative divisions**		
**Study**	**Administrative division**	**Study**	**Administrative divisions**	**National data studies**
Du et al.	Gansu province	Wang Y. et al.	Beijing, Fujian, Gansu, Guangdong, Guizhou, Hubei, Heilongjiang, Hunan, Jiangxi, Liaoning, Shandong, Shanghai, Shanxi, Sichuan, Tianjin, and Yunnan	Luo J. et al.
Zhou et al.	Guangdong province			Qi et al.
Ma H. et al.	Hubei province			Zhang et al.
Tang et al.	Hunan province			Sun and Meng
Luo Y. et al.	Hunan province	Zheng et al.	Beijing, Guangdong, and Shanghai	Liu et al.
Cen et al.	Macau special administrative region	Liu	Beijing, Hubei, Jiangsu, Liaoning, Shandong, Shanghai, Shanxi, and Zhejiang	Wen and Zhang
Li J. et al.	Shanghai municipality			Li C. et al.
Yin et al.	Shanghai municipality	Wan et al.	Guangdong, Jiangsu, and Shandong	Mai et al.
Ji and Yu	Zhejiang province			
Ma W. et al.	Zhejiang province			

Wang Q. et al., Dai et al., and Yu et al. are theoretical studies.

The bulk of the studies employed quantitative methodologies, while four studies were distinguished by their use of qualitative approaches. Among the articles that focussed on multiple administrative divisions, Wang Y. et al., Zheng et al., Liu, and Wan et al. conducted primary data collection either through questionnaires survey or interviews. Eight studies utilized secondary data from national longitudinal studies and other administrative data. These included databases such as the China Longitudinal Aging Social Survey data as in Luo J. et al., China Civil Affairs Statistical Data as in Zhang et al., Chinese Longitudinal Health Longevity Survey as in Sun and Meng, Liu et al., and Mai et al. Two studies Qi et al. and Wen and Zhang utilized data from China Health and Retirement Longitudinal Study. Li C. et al. utilized data from the Living Conditions of China's Urban and Rural older persons.

### Studies focussing on more than one administrative division

Mai et al. utilized national longitudinal data to analyze factors associated with the access to healthcare for older adults living with limited activities. The authors argue that access to healthcare services for older adults is mainly related to enabling factors such as economic status, affordability for daily life and geographical region. Being a study utilizing national data, the findings hold relevance to policies to improve geographic reach of aged care services and increasing health insurance reimbursement rates. Interestingly, the authors also call for more involvement of family members/informal carers in the provision of older adult care – the traditional model for caring elders which was deemed unsustainable in the Lancet article mentioned earlier (2). Li C. et al. utilized secondary data from the Living Conditions of China's Urban and Rural older populations to analyze the healthcare needs and differences across four administrative divisions in China (Jiangsu, Zhejiang, Beijing and Shanghai). The study concludes that preexisting medical conditions, community aged care services, education and marital status can influence the demand for aged care in the divisions studied. Additionally, the study also notes that the use of assistive technology such as smart wear use varied across different population groups, and community care can have a greater impact to older adults health. Ma W. et al., through their cross-sectional study including 3 provinces in China (Guangdong, Jiangsu and Shandong), examined participation and autonomy among adults with stroke and hypertension in home/community-based service provision. The authors call for improved community care and an enhanced role for family physicians to promptly identify low levels of social participation. This will enable prompt identification of factors that affect social participation

Utilizing qualitative research methods, Liu examined the issue of local governments' purchase of older adult care services in China. Zheng et al. focused on survey instrument development, and collected primary data from three hospitals in China, examining psychometric properties and validating the tool measuring self-care for older adults. The study by Wang Y. et al. looked at multiple regional populations across China to examine awareness of prostate cancer among older adults. Identifying the awareness levels for prostate cancer to be very low at 38%, the study called for public health programs and improving access to prostate cancer screening.

Luo Y. et al. utilized the 2014 China Longitudinal Aging Social Survey data to examine the role of informal carers (children of older adults) in the provision of financial support for rural older adults. The study identified that higher the level of financial support the mental health of rural older adults is higher. Zhang et al. utilized data from the China Civil Affairs Statistical Year book and other relevant health departments to analyse the spatial distribution of resources at long term care facilities in China. They argued the need for regional policy initiatives, particularly highlighting the development of human resources. Sun and Meng analyzed longitudinal data from the Chinese Longitudinal Health Longevity Survey and provided insights toward the role of informal caregivers. The study identified that a majority of older adults had positive attitude toward caregivers willingness to care and the care they received.

Qi et al. utilized data from the China Health and Retirement Longitudinal Study to explore the association between digital health care reform and health inequity for older adults. Though an interesting quasi experiment, they identified that digital health care reform played a vital role in diminishing health inequity and fostering inclusive growth in public health. Liu et al. utilized longitudinal data from the Chinese Health Longevity Survey and used a life course theory approach to access the impact of childhood health services on healthy life expectancy of older people. They called for improving supply chain systems and social security expansion for aged care services. Wen and Zhang explored how intergenerational support affects the oral healthcare of older Chinese adults and provide evidence for improving the oral health of the older adults in an aging society. Utilizing longitudinal data from the China Health and Retirement Longitudinal study, they stressed on the crucial role of intergenerational support and the necessity of creating an age-friendly dental care system.

### Studies focussed on a single administrative division

Li C. et al. conducted a survey of family caregivers and their disabled older relatives in Shanghai to understand the preferences of family members to care for older relatives. They identified that sense of responsibility and quality of caregiver-care recipient relationship had greater impact on care. A study by Yin et al. assessed the differences in demographic characteristics, symptoms, lifestyles, and disease comorbidities of COPD patients. Du et al. conducted a large community-based cross sectional study on knowledge of eye care utilization among 50+ older adults in 73 rural villages of Qingcheng country, Gansu province. While a better understanding of cataracts was significantly associated with cataract screening, the authors identified a misunderstanding of cataract treatment costs and timings in the rural areas. Health education activities and public health program were considered vital.

Zhou et al. investigated 627 older adults from community health centers, nursing homes and hospitals at Guangzhou province to explore perceptions on family care, depression, and quality of life. Interestingly the authors argued that family care was positively associated with purpose in life and could contribute to promoting mental health in older adults. Ma H. et al. in their study at Hubei province, looked at the design of long-term care insurance by examining the preferences of personalized needs for older adult residents. They identified that residents have a higher preference for long term care insurance and home and community-based care. Tang conducted a study on social support and mutual help needs including rural population in four regions of Hunan province. Surveying a sample of over 2000 older adults, the authors identified the importance of developing rural mutual support aged care services, and encouraged both young and older adults to join volunteer services on mutual support groups. Luo J. et al. examined perceptions of paid caregivers toward fall prevention among older adults in Hunan province, concluding that enhanced communication and cooperation were vital in falls risk assessment, prevention and management.

Cen et al.'s study from Macao, China, explored the implementation of outreach specialist program for nursing home residents. Examining data from 49 qualitative semi structured interviews of health care professionals, they argued that outreach specialist programs enhanced quality of care for nursing home residents. They also argued that cross-sector interdisciplinary collaboration and efficient data sharing and communication play a crucial role in ensuring success of the program. Li J. et al. conducted a survey of family caregivers and their disabled older relatives in Shanghai to identify the family caregiver's willingness to care. Interestingly the authors argued that reciprocal altruism presented as the quality of caregiver-care recipient relationship had a significant positive impact on family caregivers' willingness to care. Yin et al.'s study from Shanghai assessed if spirometry screening was required for chronic obstructive pulmonary disease, and newly diagnosed patients showed few differences compared with the general population and more attention was required on presenting symptoms, along with new therapies.

Ji and Yu study measured the level of older adult care service supply in Zhejiang province from 2010 to 2019.The study provided valuable insights for policy formulation in developing countries within the Asia-Pacific region and beyond, aiming to enhance the supply capacity of older adult care services and optimize the spatial distribution of resources in this domain. Ma W. et al. in their study based at Zhejiang province investigated the current knowledge of Alzheimer's disease among community health service staff. The authors argued that community health service staff had limited knowledge of Alzheimer's disease, particularly on identifying symptoms and on caregiving approaches. They proposed well developed staff training programs on Alzheimer's disease.

Three studies by Wang Q. et al., Dai et al., and Yu et al. were theory building studies. Wang Q. et al. used evolutionary gaming models to examine the performance of governments and describe the optimal path for the government to govern the older adult care service market. Dai et al., through an online Delphi study involving 198 older adults, examined the older adult care's smart supply chain, and argued the necessity to improve supply chain system as well as social security for aged care be expanded.

## Key messages and future directions

Our issue makes the case for newly emerged evidence that helps to draw a picture of the current development and investment and future direction of aged care services in China. First, family members or informal carers continue to play a major role in the provision for care for older adult, which was more visible in studies coming from regional areas of China. Studies indicated that the quality of caregiver-care recipient relationship have greater impact on care, and family care in general and improved financial contribution by children have a positive effect on promoting mental health of older adults. Second, there exists an undeniable preference for home care and community-based models witnessed from research arising both in urban as well as regional provinces. A number of studies have identified the importance of improving access and availability of services to reach homes in regional areas and reducing inequality of service provision. Integrated service provision and connecting home care personnel with the mainstream health and hospital care offers avenue to bridge differences. Third, smart technologies are seen as an avenue to reduce health care inequalities and bridging the gap between workforce availability and access to services. Fourth, the role of educational and training programs for older adults, caregivers as well as health professionals on various conditions, diseases and services was considered important. Awareness campaigns as well as screening programs were argued to improve the prevention and management of several chronic conditions. Finally, a few successful models of care for older adults have been identified in the work. This includes intergenerational care, mutual support groups and importance of volunteering. Additionally, specialist outreach models for aged care and the adoption of interdisciplinary approaches have also emerged as vital in providing services aligned with the needs and demands of older adults.

## Author contributions

MB: Conceptualization, Writing – original draft, Writing – review & editing. AS: Conceptualization, Writing – review & editing. ZL: Conceptualization, Writing – review & editing.
